# Eating habits and the desire to eat healthier among patients with chronic pain: a registry-based study

**DOI:** 10.1038/s41598-024-55449-z

**Published:** 2024-02-27

**Authors:** Huan-Ji Dong, Katherine Brain, Max Olsson, Elena Dragioti, Björn Gerdle, Bijar Ghafouri

**Affiliations:** 1 Pain and Rehabilitation Center, and Department of Health, Medicine and Caring Sciences, Linköping University, 58185 Linköping, Sweden; 2 School of Health Science, College of Health, Medicine and Wellbeing, University of Newcastle, Callaghan, NSW 2308 Australia; 3 Hunter Integrated Pain Service, Newcastle, NSW 2300 Australia; 4 Research Laboratory Psychology of Patients, Families & Health Professionals, Department of Nursing, School of Health Sciences, University of Ioannina, 45500 Ioannina, Greece; 5 Department of Neurosurgery and Pain Rehabilitation, Skåne University Hospital, 22185 Lund, Sweden

**Keywords:** Risk factors, Neurological disorders, Nutrition disorders

## Abstract

Healthcare professionals often meet pain patients with a poor nutritional status such as obesity, unhealthy dietary behaviors, and a suboptimal dietary intake. A poor nutritional status may play a significant role in the occurrence, development, and prognosis of chronic pain. This study investigated eating habits in a specialized pain rehabilitation center using data (N = 2152) from the Swedish quality registry for pain rehabilitation during the period 2016–2021. Patients answered a lifestyle questionnaire regarding their eating habits and desire to modify their lifestyle. The mean (SD) patient age was 46.1 (14.6) years, with 24.8% classified as obese. Suboptimal eating habits included irregular mealtimes (27.2%), weekly consumption of fast-food (20.3%) and nearly daily consumption of confectionery (33.3%). Approximately 20% (n = 426) reported a desire to eat healthier. Frequent confectionery intake (Odds ratio [OR] 1.23, 95% Confidence Interval (CI) 1.04–1.47) and fast-food consumption (OR 1.58, 95% CI 1.24–2.02) increased the likelihood to desire healthier eating. Younger patients (18–29 years), those classified as obese, and those with more extended spatial pain were more likely to express a desire to eat healthier. Eating habits should be addressed in pain management and interdisciplinary pain rehabilitation teams are encouraged to provide nutritional care tailored to the patient's needs.

## Introduction

Chronic pain, as defined by the International Association for the Study of Pain (IASP), is pain that persists or recurs for more than 3 months^1^. Chronic pain is associated with 75% of the global years lived with disability and affects approximately 20% of adults worldwide^1,2^. Evidence-based treatment for chronic pain incorporates the biopsychosocial approach, taking into consideration biomedical, psychological, and social factors that influence chronic pain^3,4^. This approach also includes lifestyle factors such as physical (in)activity, exercise, sleep, stress, and nutrition/diet^4,5^. All of which have a bidirectional association with pain; for example, better nutrition is associated with better pain outcomes and poor nutrition is associated with poor pain outcomes^6^. Recently, IASP recognized the importance of optimizing one’s dietary intake and encourages clinicians to address nutritional care in pain management^7^.

The role of nutrition in overall health is well established^8^ and its importance in evidence-based chronic pain management is increasingly being acknowledged^9^. Biomedically, pain is associated with several mechanisms that can be modulated by diet, including oxidative stress, inflammation, alterations in the gut microbiome, disturbances in glucose and lipid metabolism, and central nervous system alterations^10–12^. Additionally, psychosocial stresses like depression and isolation are prevalent among people with chronic pain^13,14^, leading to changes in eating behaviors and low diet quality^15–17^. Diet quality is a measure with refined scoring methods (i.e. diet quality indexes) that demonstrates how closely aligned eating patterns are to national dietary guidelines^18^. Diet quality also considers the diversity of healthy options consumed in the core food groups e.g., vegetables, fruits, whole grains, meat, dairy, and their plant-based alternatives. Higher diet quality has been consistently linked to better quality of life and health outcomes^18–20^. Conversely, low diet quality characterized by a limited variety of healthy foods and excessive consumption of energy-dense, nutrient-poor processed foods is associated with poorer health outcomes^21^. In western societies, individuals’ eating behaviors are challenged by convenient and ultra-processed foods that are typically low in nutritional value, thereby lowering diet quality.

Understanding patients' eating behaviors is crucial in informing interventions^22–24^. Various factors, including socio-demographics, attitudes toward food and health, psychological status, and social and environmental influences, can impact diet quality, eating habits and the desire to change behaviors^24–26^. Many studies have highlighted the prevalence of poor nutrition among individuals with chronic pain. For instance, a clinical audit of patients attending a chronic pain service in Australia revealed that most patients had a suboptimal diet quality score, indicating a need for dietary improvement^27^. Other studies have reported low fruit and vegetable intake and excessive consumption of unhealthy fats among individuals with chronic pain^28,29^.

Despite this growing body of evidence, there is a lack of any recent, large-scale surveys investigating eating habits among patients with chronic pain, particularly those that have been conducted in a clinical setting. To address this gap, our study aimed to investigate the eating habits and explore factors associated with the desire to adopt healthier dietary behaviors among individuals with complex chronic pain^30^ referred to a specialized pain rehabilitation center in Sweden. By examining eating patterns and factors influencing patients' motivation to make healthier dietary choices, healthcare professionals can tailor rehabilitation goals and content to provide patient-centered care^31,32^.

## Methods

### Subjects

The study included patients referred from primary care or other specialist care (e.g. orthopedics, rheumatology) in the Östergötland County to the specialized Pain and Rehabilitation Centre, Linköping University Hospital between August 2016, and December 2021. Common chronic pain diagnoses were widespread pain including fibromyalgia, neck pain, lower back pain and hypermobile Ehler-Danlos syndrome. At a specialist care level, patients had non-malignant chronic pain (≥ 3 months) with complex clinical presentations including psychosocial stress. Common psychosocial stresses include anxiety, depression, and social isolation. Patients also frequently presented with physical and/or functional impairment impacting their ability to work and contribute to society.

### Swedish quality registry for pain rehabilitation (SQRP)

The Swedish quality registry for pain rehabilitation (SQRP) was established in 1995, collecting patient-reported data on socio-demographics, pain aspects, psychometric data, physical disability, and quality of life^33,34^. Several self-administered questionnaires (see below) have been included in parallel with the development of pain research, for example, data on body weight and insomnia were included in 2016^35,36^. Pain and Rehabilitation Centre, Linköping University Hospital is one of the 42 pain-rehabilitation clinics (> 90% pain clinics or units) that collect SQRP data in Sweden. Approximately two weeks prior to their first consultation with a physician, patients received a postal letter with these questionnaires, and were asked to complete them before their appointment. Written and signed informed consent was obtained for SQRP data collection. The questionnaire is a mandatory clinical tool, however approximately 10% of patients do not sign consent or return to the questionnaire and therefore their data is not included in the registry. Based on our clinical experience, this may be due to language barriers, low health literacy or cognitive impairment. An administrator in the department transferred the data into a local database with a software program provided by the registry. The variables and instruments included in the questionnaires were mandatory for the clinical specialist departments registering their data with the SQRP. SQRP also collects data from patients at other timepoints during the rehabilitation periods. For this study, we only investigated the data that was collected prior to the patient’s consultation with a physician (i.e. baseline data, N = 2152).

### Ethics

Verbal and written information about this study using SQRP data was given to all the participants and written informed consent was thereafter obtained from all the participants. The study was conducted in accordance with the Helsinki Declaration. The study was approved by the Swedish Ethical Review Authority (dnr: 2021-02811).

### Background factors

Socio-demographic factors such as gender, age (years), country of origin (Sweden, Nordic, European or other), and education (primary school, secondary school, or university/college) were extracted from SQRP. Economic status was determined based on the specific item from the Life Satisfaction questionnaire LiSAT-11. This captured the patient’s perceived satisfaction with the economy (LiSAT-economy)^37^. Six possible answers were given: 1 = very dissatisfying; 2 = dissatisfying; 3 = fairly dissatisfying; 4 = fairly satisfying; 5 = satisfying; and 6 = very satisfying.

Patients self-reported body weight and height, and body mass index (BMI = weight (kg)/height (m)^2^) was calculated. The BMI category is defined according to the World Health Organization (WHO): BMI < 18.5 = Underweight; BMI 18.5–24.9 = Normal Weight; BMI 25–29.9 = Overweight; and BMI ≥ 30 = Obesity^38^.

### Pain characteristics

Pain intensity was defined as the average pain intensity during the previous week using a numeric rating scale from 0 to 10; 0 being no pain and 10 being the worst pain imaginable.

Patients reported the date when they first started experiencing their current pain. Pain duration was then calculated based on this date and the number of years since the onset of pain. The variable was also coded as a binary variable (pain lasting for 5 years or more = 1, pain lasting less than 5 years = 0).

Spatial pain was examined using a body map with 36 predefined anatomical areas (18 on the front and 18 on the back of the body) and patients were asked to indicate their pain locations. The number of pain locations were summarized, and this variable was denoted as the Pain Region Index (PRI)^39^.

### Hospital anxiety and depression scale (HADS)

The HADS is a 14-item questionnaire to measure anxiety (HADS-A, 7 items) and depression (HADS-D, 7 items), with a higher score indicating a higher possibility of anxiety and/or depression. Levels ≥ 11 (possible range: 0–21 for each subscale) indicates a definite case for anxiety or depression. An overall score was also provided to measure emotional distress^40^.

### Eating habits: dietary, tobacco, and alcohol consumption

For the identification of unhealthy lifestyle behaviors, a lifestyle questionnaire was constructed in the registry based on a report from the Swedish National Board of Health and Welfare^41^. This study included questions regarding dietary behaviors (5 items), tobacco consumption (2 items) and alcohol consumption (2 items). Dietary behaviors consisted of the following items: (1) regular mealtimes (always irregular, seldom regular, tried but not regular, usually regular, or always regular), consumption of (2) vegetables (at least 2–3 times per day, once per day, several times per week, or seldom/ never), (3) fruits (4–5 servings per day, 1–2 servings per day, sometimes, or almost never), (4) fast-foods such as pizza, kebab, hotdogs, etc. (never, seldom, 1–2 times per week, several times per week, every day), and (5) confectionary (never, once per week, several times per week, sometime every day, several times every day). Fruit and vegetable intake was combined to yield a total score ranging from 0 to 8. A low score indicated a low intake, and high score indicated a high intake. Tobacco use included (1) moist snuff consumption and (2) cigarette smoking. Current consumption of moist snuff and/or cigarette smoking was denoted as a tobacco user. Data on both the frequency and quantity of alcohol consumption was collected. The frequency was listed as ‘every day’, ‘1–2 times a week’, ‘every other week’, ‘once a month’, or ‘no drinking’. The frequency was rated on a five-point Likert scales (0 = ‘no drinking and 5 = ’every day’). The amount of consumption on one occasion was counted as ‘1–2 glasses of wine or 4 centiliters of spirits’, ‘2–3 glasses of wine or 8–12 centiliters of spirits’, or ‘more than one bottle of wine and 12 centiliters of spirits’. A grade from 0 to 2, with higher scores indicating a higher amount consumption. Patients who desired (PD) to eat healthier were ones that selected it as a priority when they were asked whether they wanted to modify their current lifestyle and chose one or more of the following areas to prioritize: (1) healthier eating, (2) increasing physical activity, (3) achieving weight loss, (4) smoking cession, (5) decreasing alcohol consumption and (6) none of the above. Patients who did not desire (PND) to eat healthier were respondents who did not choose it as a priority.

### Statistical analysis

All statistical analysis were performed using IBM SPSS statistics (version 28.0). Descriptive data were presented using the mean and standard deviation (Mean ± SD), the mean with 95% confidence intervals (CI), or the number with the percentage, where appropriate. The desire to eat healthier was coded as a binary variable (0 = no, 1 = yes). For comparison of patients who desired healthier eating with those who did not, the χ^2^ test for categorical variables, Mann–Whitney *U* test for non-normally distributed ordered categorical variables and Student’s *t* test for normally distributed variables were used. Correlation analysis between eating habits and variables of interest was performed using Spearman’s Rho correlation test. A *p* value below 0.05 was considered statistically significant. We performed univariate and multivariate logistic regressions (likelihood ratio forward measure) to identify possible predictors for patients to desire healthier eating. The results were expressed as odd ratios (OR) with 95% confidence intervals (95% CI), indicating each predictor increased (OR > 1) or decreased (OR < 1) odds for patients to desire healthier eating. The selection criteria of explanatory variables to enter in the multivariate regression model was based on a relaxed type I error (*p* ≤ 0.25) in the univariate analyses^42^. Each variable was carried forward, and the variable with highest *p* value was removed (*p* < 0.05 or *p* ≥ 0.1 for entry or removal, respectively). The Hosmer and Lemeshow test was used to examine the goodness of fit (*p* > 0.05). Logistic regression analyses as default use listwise deletion to handle the missing cases. To examine if missing data can lead to biased results, we also performed a sensitivity analysis where we used multiple imputation to handle the missing data as a comparison to the results based on ‘real completed cases’ (see Supplementary Material).

## Results

### General characteristics of the study population

As shown in Table 1 (N = 2152), many of the patients were in middle age (mean 46.1 ± 14.6, 56.8% aged 30–54 years), women (71.8%) and born in a Nordic country (84%). One in five patients had a university/college education and less than 30% were satisfied with their economic status. More than half of the patients were overweight or obese (mean BMI 27.2 ± 5.6) and reported long pain duration (≥ 5 years). One in four (24.8%) was classified as obese (BMI ≥ 30 kg/m^2^). High pain intensity (mean NRS-7d 7.1 ± 1.8), wide pain spreading (spatial extent of pain, PRI 14.4 ± 9.0) and mild depression and/or anxiety levels (mean HADS-D 8.6 ± 4.7 and mean HADS-A 8.8 ± 5.0) were also found in this study population. Approximately one in three (32.6%) were classified as clinically emotionally distressed. In comparison to PND, PD (n = 426, 19.8%) were younger, less satisfied with economic status, had a higher BMI, longer pain duration, and reported more extended spatial experience of pain and more severe emotional distress (p < 0.001 ~ 0.05).

**Table 1:** Characteristics of the study population, n (%) if not otherwise stated.

	All patients, N = 2152^1^	Patients who desired to eat healthier (PD), n = 426	Patients who did not desire to eat healthier (PND), n = 1720^2^	*P-*value (*PD* vs *PND*)
Age, mean ± SD	46.1 ± 14.6	42.0 ± 14.0	47.1 ± 14.6	** < 0.001**
18–29 years	339 (15.8)	98 (23.0)	241 (14.0)	** < 0.001**
30–54 years	1222 (56.8)	250 (58.7)	966 (56.2)	
55 + years	591 (27.5)	78 (18.3)	513 (29.8)	
Female gender	1545 (71.8)	317 (74.7)	1223 (71.1)	0.174
Country of birth				0.622
Nordic country	1746 (84)	344 (84.5)	1396 (84.7)	
Other European country	85 (3.9)	65 (3.9)	20 (4.9)	
Outside Europe	231 (10.7)	43 (10.6)	188 (11.4)	
University/college	495 (23)	402 (27.0)	92 (24.7)	0.380
LiSAT- economy, satisfied	575 (29.8)	89 (23)	486 (31.6)	**0.001**
BMI, mean ± SD	27.2 ± 5.6	28.0 ± 6.4	27.0 ± 5.3	**0.004**
Underweight	40 (1.9)	10 (2.5)	29 (1.8)	** < 0.001**
Normal weight	732 (37.0)	137 (34.9)	594 (37.6)	
Overweight	673 (31.3)	109 (27.7)	563 (35.6)	
Obesity	534 (24.8)	137 (34.9)	394 (24.9)	
Pain duration, years, mean (95% CI)	9.7 (9.2–10.2)	10.4 (9.3–11.5)	9.6 (9.0–10.1)	**0.020**
≥ 5 years since pain debut	966 (52.5)	206 (58.2)	760 (51.1)	**0.016**
Pain intensity (NRS-7d), mean ± SD	7.1 ± 1.8	7.1 ± 1.7	7.1 ± 1.8	0.355
Pain regional index, mean ± SD	14.4 ± 9.0	16.5 ± 9.1	14.4 ± 9.0	** < 0.001**
HADS-A, mean ± SD	8.8 ± 5.0	9.7 ± 5.1	8.5 ± 4.9	** < 0.001**
HADS-D, mean ± SD	8.6 ± 4.7	9.0 ± 4.4	8.5 ± 4.7	**0.026**
HADS-total, mean ± SD	17.3 ± 8.7	18.6 ± 8.5	17.0 ± 8.7	** < 0.001**
Anxiety indicated by HADS-A	742 (34.5)	188 (46.4)	550 (33.9)	** < 0.001**
Depression indicated by HADS-D	680 (31.6)	153 (37.9)	525 (32.2)	**0.032**
Emotional distress indicated by HADS-total score ≥ 22	661 (32.6)	161 (39.9)	500 (30.8)	** < 0.001**

*SD* standard deviation, *CI* confidence interval, *LiSAT* life satisfaction questionnaire, *BMI* body mass index, *NRS-7d* numeric pain scale during the last 7 days, *HADS* hospital anxiety and depression scale, *HADS*-*A* HADS-anxiety, *HADS-D* HADS-depression.

^1^Missing cases in the variables of interest included in the supplementary document; ^2^Missing data = 6, referring to those who left the answer blank.

Significant values are in bold.

### Eating habits and their correlations to other factors

**Figure 1:**
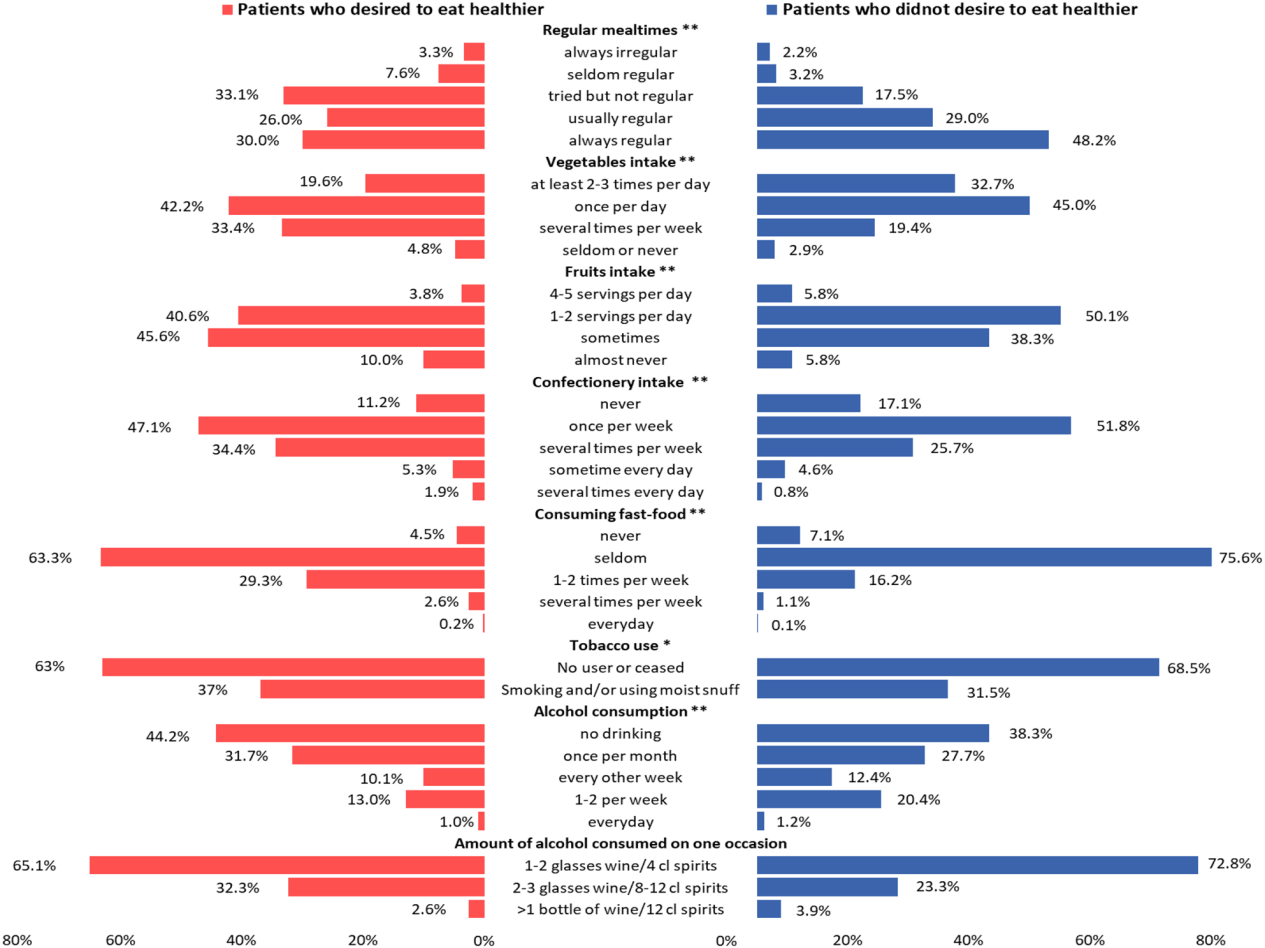
Differences of eating habits between patients who desired to eat healthier and those who did not. The X axis represents the proportion of patients’ choices in each category of eating habits. Red bars show the proportion of patients who desired to eat healthier and blue bars represent patients who did not desired to eat healthier. Chi-square test, *P < 0.05, **P < 0.01.

A summary of self-reported dietary, smoking and alcohol habits is listed in Fig. 1. A little over one-quarter, 27.2%, of patients had irregular mealtimes (i.e. always irregular, seldom regular, and tried but failed to keep regular mealtimes). PD had more frequent irregular mealtimes than PND (t = − 8.01, *p* < 0.001) and the percent of patients having irregular mealtimes almost doubled in PD (44%) than that in PND (22.9%). Second, PD had less frequent vegetable (t = 6.94, *p* < 0.001) and fruit intake (t = 4.64, *p* < 0.001) than PND. Those who did not consume vegetables or fruit on a daily basis made up 28.4% in PD, almost doubled compared to PND (15.4%, χ^2^ = 38.07, *p* < 0.001). Third, one in five (20.3%) reported weekly or daily fast-food consumption (1–2 times per week, several times per week or every day). One in three (33.3%) reported that they nearly daily consumed confectionary (from several times per week to several times every day). PD reported higher consumption of confectionary (t = 4.27, *p* < 0.001) and fast food (t = 5.97, *p* < 0.001) compared to PND. Fourth, only a slight significant difference was found in tobacco use between the two groups (χ^2^ = 4.60, *p* = 0.032), that a slightly higher proportion of PD were currently smoking and/or using moist snuff. Finally, PD reported lower frequency of alcohol consumption (t = − 3.94, *p* < 0.001) than PND. There was no statistical difference between the two groups (t = 1.63, *p* = 0.052) when it came to consuming alcohol on any single occasion. A majority (71.3%) reported drinking 1–2 glasses of wine or 4 centiliters of spirits and only few patients (3.6%) reported drinking a bottle of wine and 12 centiliters of spirits on any one occasion.

Some significant correlations between eating habits and other variables of interest are shown in Table 2. Given a large study population, Spearman’s rho was low (|r|= 0.05 ~ 0.25), indicating weak correlations. Regularity of mealtimes was significantly correlated to pain aspects (NRS-7d, PRI, and pain duration), HADS-total and the subscales, obesity, and socio-demographic factors (age, gender, education, and LiSAT-economy). Likewise, frequency of vegetable and/or fruit intake was correlated to all variables except PRI and pain duration. Three variables -NRS-7d, age and gender—were significantly correlated to all variables of suboptimal eating habits.

**Table 2:** Correlations between patients’ eating habits and other characteristics (i.e. pain aspects, emotional distress, weight status and socio-demographics).

	Regularity of mealtimes	Frequency of vegetables and fruits intake	Frequency of confectionary consumption	Frequency of fast-food consumption	Tobacco Use	Frequency of alcohol consumption	Amount of alcohol consumed on one occasion
Pain intensity (NRS-7d)	− 0.097**	− 0.074**	− 0.052*	− 0.061**	0.096**	− 0.187**	− 0.145**
Pain regional index	− 0.159**	− 0.008	0.044	0.032	− 0.004	− 0.139**	− 0.136**
Pain duration	− 0.052*	− 0.025	0.012	− 0.017	0.053*	− 0.016	− 0.026
HADS-total score	− 0.292**	− 0.147**	0.039	0.027	0.141**	− 0.167**	− 0.111**
HADS-A	− 0.261**	− 0.128**	0.036	0.039	0.141**	− 0.144**	− 0.072**
HADS-D	− 0.262**	− 0.134**	0.034	0.010	0.113**	− 0.156**	− 0.129**
BMI	− 0.022	− 0.066**	0.055*	0.064**	− 0.010	− 0.053*	− 0.022
Obesity category	− 0.052*	− 0.070**	0.039	0.050*	− 0.010	− 0.068**	− 0.019
Age	0.193**	0.118**	− 0.093**	− 0.163**	− 0.125**	0.133**	− 0.075**
Gender	− 0.071**	0.137**	0.082**	-0.099**	− 0.124**	− 0.072**	− 0.101**
University/college education	0.125**	0.187**	− 0.052*	− 0.061**	− 0.204**	0.085**	0.011
LiSAT-economy	0.253**	0.112**	0.001	− 0.015	− 0.152**	0.172**	0.079**

*NRS-7d* numeric pain scale during the last 7 days, *HADS* hospital anxiety and depression scale, *BMI* body mass index, *LiSAT* life satisfaction questionnaire.

Binary variables: pain duration (0 =  < 5 years, 1 = at least 5 years since pain debut), obesity category (0 = non-obese, 1 = obese), female gender (0 = male, 1 = female), university/college education (0 = no, 1 = yes), LiSAT-economy (0 = not satisfied, 1 = satisfied), tobacco use (0 = no, 1 = yes). All other variables were presented as numerical or ordinal variables. *p < 0.05, **p < 0.01.

Significant values are in bold.

### Factors associated with patients’ desire for healthier eating.

Through the univariate logistic regression analysis (Table 3), the desire to eat healthier was primarily related to sub-optimal eating habits such as an increased consumption of confectionary (OR 1.33, 95% CI 1.17–1.51) and fast-food (OR 1.87, 95% CI 1.54–2.26). Eating habits such as regular mealtimes and a high intake of fruit and/or vegetables, were negatively associated with the patients’ desire for healthier eating (OR 0.66–0.73, *p* < 0.001). This remained significant in the multivariate logistic regression model when other variables were also included. Tobacco and alcohol consumption only showed statistical significance in the univariate analysis.

**Table 3:** Binary logistic regression (forward LR) – factors associated with patients’ desire for healthier eating.

	Variables associated with patients’ desire for healthier eating	
	Univariate analysis [OR, 95% CI]	Multivariate model [OR, 95% CI]^1^
Eating habits		
Regularity of mealtimes	**0.66 (0.60–0.73)**	**0.75 (0.66–0.87)**
Frequency of vegetables and fruits intake	**0.46 (0.36–0.59)**	**0.83 (0.74–0.94)**
Frequency of confectionary consumption	**1.33 (1.17–1.51)**	**1.21 (1.03–1.44)**
Frequency of fast-food consumption	**1.87 (1.54–2.26)**	**1.55 (1.21–1.98)**
Tobacco Use (0 = no, 1 = yes)	**1.28 (1.02–1.60)**	Not included
Frequency of alcohol consumption	**0.83 (0.76–0.92)**	Not included
Amount of alcohol consumed	1.24 (0.96–1.59)	Not applied
General characteristics		
Age (55 + y, reference category)		
18–29 years	**2.77 (1.98–3.89)**	**1.87 (1.20–2.91)**
30–54 years	**1.75 (1.32–2.32)**	1.43 (0.99–2.07)
Gender (0 = male, 1 = female)	1.16 (0.91–1.48)	Not included
Country of birth (nordic country, reference category)		Not applied
Any other European country	1.25 (0.75–2.09)	
Outside Europe	0.93 (0.65–1.32)	
University/college education (0 = no, 1 = yes)	0.89 (0.68–1.16)	Not applied
LiSAT-economy (0 = not satisfied, 1 = satisfied)	**0.65 (0.50–0.84)**	Not included
Obesity category (0 = non-obese, 1 = obese)	**1.51 (1.19–1.93)**	**1.37 (1.02–1.84)**
Pain intensity (NRS-7d)	1.01(0.95–1.08)	Not applied
Pain duration (0 = < 5 years, 1 = at least 5 years since pain debut)	**1.33 (1.06–1.68)**	Not included
Pain regional index	**1.03 (1.01–1.04)**	**1.02 (1.01–1.04)**
Emotional distress (0 = HADS-total score < 22, 1 = HADS-total score ≥ 22)	**1.49 (1.12–1.86)**	Not included

Significant ORs (CIs) are given in bold.

*LiSAT* life satisfaction questionnaire, *NRS-7d* numeric pain scale during the last 7 days, *OR* Odds ratio; *CI* confidence interval.

Not included: variable selected in the regression model but not included in the final step via LR Forward method.

Not applied: variable not selected in the regression model due to low significant level (*p* > 0.25) in the univariate regression model.

^1^Nagelkerke R^2^ = 0.121.

General characteristics were also examined in the logistic regression models. In the multivariate regression model, patients aged 18–29 years (OR 1.87, 95% CI 1.20–2.91), who fell into the obese BMI category (OR 1.37, 95% CI 1.02–1.84), and who suffered more spatial extent of pain (OR 1.02, 95% CI 1.01–1.04) were more likely to report the desire for healthier eating. In the univariate analysis, patients who dissatisfied with their personal economic status, had a long pain duration, or had high levels of emotional distress were more likely to report the desire for healthier eating However, these effects were no longer significant in the latter multivariate regression model.

## Discussion

In this registry-based study at a specialized pain rehabilitation center, we identified some common suboptimal eating habits in patients with chronic pain, such as irregular mealtimes, weekly or daily consumption of confectionary and fast-food. These habits were significantly associated with the patients’ desire to eat healthier. Moreover, we found that patients who were younger, fell into the obese BMI category and suffered emotional distress were more likely to desire healthier eating. These findings indicate there is a need and want for lifestyle interventions, especially nutrition support, among patients with chronic pain in a specialist pain and rehabilitation center, which seems to have been a neglected aspect in the past Interdisciplinary Pain Rehabilitation Program (IPRP)^30,43^.

In everyday clinical practice, lifestyle questionnaires can be used to identify suboptimal eating habits. In this study, we selected several items focusing on their modifiability and potential post-modification benefits. For example, irregular mealtimes has been correlated with mental health issues^44^ or severe gastrointestinal symptoms^45^. Regarding food choices, the questions in our questionnaire corresponded to the Nordic Dietary Recommendations (NNR) for increased intake of vegetables and fruit and limited consumption of discretionary foods and drinks^46^. The literature has identified that smoking is related to a high prevalence of chronic pain and higher pain intensity^47^. Likewise, alcohol has been reported as a common coping mechanism for people suffering from chronic pain^48^. Findings from these previous studies indicate the importance of screening eating habits. Although we reported weak correlations between pain intensity and patients’ eating habits in this study population, we were aware of the complex interplay between pain intensity and other biopsychosocial factors. This should be taken into account in further research, especailly in nutritional intervention studies. Chronic pain has sometimes been regarded as a lifestyle disease in pain research^5,49^. To optimize the success of lifestyle interventions, it is necessary to address patients’ needs and wants, so that the intervention aligns with their motivation and engagement and reflects patient-centered care. Our regression model suggested that the patients with suboptimal eating habits want to improve their dietary intake and habits. However, we cannot definitively conclude the reasons for their desire or understand if patients’ awareness (or not) of the relationship between pain and eating habits impacted on their desire. Low health literacy is one factor that may be a potential barrier impacting patients understanding and awareness of the relationship between nutrition and chronic pain or overall health^50^. It is essential that clinicians are aware that while patients may desire change, they may not know why, or how, to change. Health care professionals are well placed to address these potential gaps using education and behavior change strategies^24^.

Lifestyle factors have key roles in the development of chronic pain^5^. In addition to physical activity and sleep disorders which are already well addressed in pain management^51,52^, attention to nutrition support is needed^7,11^. However, there are several challenges that may prevent lifestyle change. Recent studies on patients with lower back pain showed negative results after lifestyle interventions^53,54^. The authors postulate that poor adherence may explain this outcome. Tailored and person-centered approaches in nutrition support are essential to overcome this barrier^11,12^. It is also important to identify which lifestyle factors patients want to change to optimize their motivation and help clinicians to improve future tailored IPRP targeting these patients. Some patient characteristics, such as age, BMI and emotional distress (scoring high HADS), should also be considered alongside underlying motivations (i.e. sub-optimal eating habits). To consider patients’ desire about lifestyle changes is also consistent with the previous research about patients’ expectation in customizing their pain rehabilitation^55^. Changing behavior and/or habits requires more than just education targeting healthy eating. Behavior change and communication strategies and techniques, such as the COM-B model (interactions of capability, opportunity, and motivation of behavior change)^56^ and Healthy Conversation Skills^57,58^ are essential to identify motivators and barriers that may help or hinder patients and facilitate change. Situational factors, self-regulation skills and contingencies may also be needed to target the patients’ goals^24^. To optimize the success and sustainability of behavior change, it is also important to focus on the role of nutrition and pain management, rather than weight loss. This is more likely to resonate with patients and prevent the negative impacts of weight stigma and highly restrictive diets which can have serious biopsychosocial effects^59^. A one-size fits all approach is not appropriate in pain rehabilitation. Nutrition assessments are required so that dietitians and health professionals can identify areas where a patient can improve their eating habits. This also allows dietitians to target personalized nutrition advice and strategies. During this process, by using behavior change communication strategies, the dietitian or health professional can also determine the patient’s willingness to change their eating habits. This should be included when tailoring IPRP for patients.

To the best of our knowledge, this study is the first to present eating habits among patients referred to pain rehabilitation clinics at specialist care. The novelty of this study is that we collected clinical data to address important lifestyle factors, which seems to be overlooked in chronic pain management. Using registry data with a large sample size, enabled us to provide practice-based evidence in pain and rehabilitation research. When patients filled out the questionnaires, they did not receive any motivational interviewing or education about nutrition’s role in pain management. Their sub-optimal eating habits and the desire for healthier eating may encourage clinicians to address nutrition care in future pain rehabilitation. This study has several limitations. Firstly, as a cross-sectional analysis, we cannot determine the causal-effect relationship. Lifestyle change takes time and needs appropriate measurement to evaluate the possible changes during the follow-up months to years. Secondly, the study population was limited to a specialist pain and rehabilitation center and there was no control group. In Sweden, patients referred to specialist care are considered to have more complex pain conditions and our results may be generalized to this patient group at specialist care level but interpretation beyond this population is limited. Patients with chronic pain in primary care may have different eating habits. Thirdly, lifestyle factors we analyzed in this study were self-reported data and pragmatically applied in clinical practice. This lifestyle questionniare was a brief screening tool for clnical assessments and lacked definitions and serve sizes for fruit and vegetables, and fast-food. This limits the ability to calcuate the amount and type of fruit and vegetables and fast food consumed. The use of a detailed qestionnaire or further indivudal interview by dietitian or nurse in the rehabiliation team would allow more specifc data to be collected. Validation of the questions for research use should also be considered. Fourth, we did not measure patients’ attitudes to food and health which can influence eating habits and the desire for behavioral change^24,25^. Patients with chronic pain may also face challenges in maintaining a healthy diet due to difficulties in shopping, meal preparation, and cooking^7^. These factors should be considered when developing future studies, especially those studies which develop, implement and test nutritional interventions for people experiencing chronic pain.

In conclusion, using real-world data and pragmatic instruments in clinical practice, we found that suboptimal eating habits were common in patients with chronic pain, such as irregular mealtimes, frequent consumption of confectionery and fast-food. Many patients reported a desire to eat healthier, highlighting the need for eating habits to be acknowledged and addressed in pain management. While patients indicated their desire to eat healthier, it is unclear if they know why nutrition is important and/or how to change their eating habits. To address patients' desire for healthier diets, we anticipate tailored lifestyle interventions to be integrated into future IPRPs targeting those patients in need.

### Supplementary Information

Supplementary Information.

## Data Availability

The datasets generated and/or analyzed in this study are not publicly available as the Ethical Review Board has not approved the public availability of these data. The data that support the findings of this study are available from SQRP (https://www.ucr.uu.se/nrs/) but restrictions apply to the availability of these data, which were used under license for the current study, and so are not publicly available. Data are however available from the corresponding author on reasonable request and with permission of SQRP research group (address: NRS, Skånes Universitetssjukhus, Smärtrehabilitering, Lasarettsgatan 13, SE 221 85 Lund, Sweden; Register holder: Marcelo Rivano Fischer, Marcelo.rivanofischer@skane.se).

## References

[1] Treede RD (2019). Chronic pain as a symptom or a disease: The IASP classification of chronic pain for the international classification of diseases (ICD-11). Pain.

[2] Global Burden of Disease (GBD) Risk Factors Collaborators (2020). Global burden of 87 risk factors in 204 countries and territories, 1990–2019: A systematic analysis for the Global Burden of Disease Study 2019. Lancet.

[3] Nicholas MK (2022). The biopsychosocial model of pain 40 years on: Time for a reappraisal?. Pain.

[4] Cohen SP, Vase L, Hooten WM (2021). Chronic pain: An update on burden, best practices, and new advances. Lancet.

[5] Nijs J (2020). Lifestyle and chronic pain across the lifespan: An inconvenient truth?. PM R.

[6] Elma O (2022). Diet can exert both analgesic and pronociceptive effects in acute and chronic pain models: A systematic review of preclinical studies. Nutr. Neurosci..

[7] Brain, K. B., T.L.; Rollo, M.; Collins, C. *The International Association for the Study of Pain (IASP). Nutrition and Chronic Pain*. https://www.iasp-pain.org/resources/fact-sheets/nutrition-and-chronic-pain/ (2020).

[8] Global Burden of Disease (GBD) Diet Collaborators (2019). Health effects of dietary risks in 195 countries, 1990–2017: A systematic analysis for the Global Burden of Disease Study 2017. Lancet.

[9] Brain K (2019). A systematic review and meta-analysis of nutrition interventions for chronic noncancer pain. J. Hum. Nutr. Diet.

[10] Nijs J (2021). Central sensitisation in chronic pain conditions: Latest discoveries and their potential for precision medicine. Lancet Rheumatol..

[11] Elma O, Brain K, Dong HJ (2022). The importance of nutrition as a lifestyle factor in chronic pain management: A narrative review. J. Clin. Med..

[12] Brain K (2021). Diet and chronic non-cancer pain: The state of the art and future directions. J. Clin. Med..

[13] Karayannis NV, Baumann I, Sturgeon JA, Melloh M, Mackey SC (2019). The impact of social isolation on pain interference: A longitudinal study. Ann. Behav. Med..

[14] Meda RT (2022). Chronic pain-induced depression: A review of prevalence and management. Cureus.

[15] Bigand T, Wilson M (2019). Overeating during painful episodes among adults with chronic pain: A preliminary study. Appetite.

[16] Emami AS, Woodcock A, Swanson HE, Kapphahn T, Pulvers K (2016). Distress tolerance is linked to unhealthy eating through pain catastrophizing. Appetite.

[17] Masheb RM, Douglas ME, Kutz AM, Marsh AG, Driscoll M (2020). Pain and emotional eating: Further investigation of the Yale emotional overeating questionnaire in weight loss seeking patients. J. Behav. Med..

[18] Wirt A, Collins CE (2009). Diet quality–what is it and does it matter?. Public Health Nutr..

[19] Sotos-Prieto M (2017). Association of changes in diet quality with total and cause-specific mortality. N. Engl. J. Med..

[20] Vajdi M, Farhangi MA (2020). A systematic review of the association between dietary patterns and health-related quality of life. Health Qual. Life Outcomes.

[21] Fanelli SM (2020). Poorer diet quality observed among US adults with a greater number of clinical chronic disease risk factors. J. Prim. Care Community Health.

[22] Gardner B (2021). Breaking habits or breaking habitual behaviours? Old habits as a neglected factor in weight loss maintenance. Appetite.

[23] Meldrum DR, Morris MA, Gambone JC (2017). Obesity pandemic: Causes, consequences, and solutions-but do we have the will?. Fertil. Steril..

[24] Van’t Riet J, Sijtsema SJ, Dagevos H, De Bruijn GJ (2011). The importance of habits in eating behaviour. An overview and recommendations for future research. Appetite.

[25] Brug J (2008). Determinants of healthy eating: Motivation, abilities and environmental opportunities. Fam. Pract..

[26] Phelan JM, Rosenkranz RR, Phelan CJ, Rosenkranz SK (2023). Holistic framework to contextualize dietary quality assessment: A critical review. Int. J. Environ. Res. Public Health.

[27] Brain K (2017). Population characteristics in a tertiary pain service cohort experiencing chronic non-cancer pain: Weight status, comorbidities, and patient goals. Healthcare (Basel).

[28] Vandenkerkhof EG, Macdonald HM, Jones GT, Power C, Macfarlane GJ (2011). Diet, lifestyle and chronic widespread pain: Results from the 1958 British Birth Cohort Study. Pain Res. Manag..

[29] Meleger AL, Froude CK, Walker J (2014). Nutrition and eating behavior in patients with chronic pain receiving long-term opioid therapy. PM R.

[30] Gerdle, B., Fischer, M. R. & Ringqvist, Å. Interdisciplinary Pain Rehabilitation Programs: Evidence and Clinical Real-World Results (2022).

[31] Elwyn G (2012). Shared decision making: A model for clinical practice. J. Gen Intern. Med..

[32] Geurts JW (2017). Patient expectations for management of chronic non-cancer pain: A systematic review. Health Expect.

[33] Gerdle B, Molander P, Stenberg G, Stalnacke BM, Enthoven P (2016). Weak outcome predictors of multimodal rehabilitation at one-year follow-up in patients with chronic pain-a practice based evidence study from two SQRP centres. BMC Musculoskelet. Disord..

[34] Nyberg V, Sanne H, Sjolund BH (2011). Swedish quality registry for pain rehabilitation: Purpose, design, implementation and characteristics of referred patients. J. Rehabil. Med..

[35] Alfoldi P, Dragioti E, Wiklund T, Gerdle B (2017). Spreading of pain and insomnia in patients with chronic pain: Results from a national quality registry (SQRP). J. Rehabil. Med..

[36] Dong HJ, Larsson B, Rivano Fischer M, Gerdle B (2019). Maintenance of quality of life improvement for patients with chronic pain and obesity after interdisciplinary multimodal pain rehabilitation—A study using the Swedish Quality Registry for Pain Rehabilitation. Eur. J. Pain.

[37] Fugl-Meyer AR, Bränholm I-B, Fugl-Meyer KS (2016). Happiness and domain-specific life satisfaction in adult northern Swedes. Clin. Rehabilit..

[38] World Health Organization (WHO). *Obesity and overweight.* http://www.who.int/mediacentre/factsheets/fs311/en/index.html (2011).

[39] Gerdle B, Rivano Fischer M, Cervin M, Ringqvist A (2021). Spreading of pain in patients with chronic pain is related to pain duration and clinical presentation and weakly associated with outcomes of interdisciplinary pain rehabilitation: A cohort study from the Swedish quality registry for pain rehabilitation (SQRP). J. Pain Res..

[40] LoMartire R, Ang BO, Gerdle B, Vixner L (2020). Psychometric properties of short form-36 health survey, EuroQol 5-dimensions, and hospital anxiety and depression scale in patients with chronic pain. Pain.

[41] The Swedish National Board of Health and Welfare (2013). Disease Prevention in the Swedish Healthcare System: Health situation, national guidelines and implementation, Indicators, Appendix (Nationella riktlinjer för sjukdomsförebyggande metoder 2011, Indikatorer, Bilaga).

[42] Stoltzfus JC (2011). Logistic regression: A brief primer. Acad. Emerg. Med..

[43] Dong HJ, Dragioti E, Rivano Fischer M, Gerdle B (2021). Lose pain, lose weight, and lose both: A cohort study of patients with chronic pain and obesity using a national quality registry. J. Pain Res..

[44] Tahara Y (2021). Association between irregular meal timing and the mental health of Japanese workers. Nutrients.

[45] Nilholm C, Larsson E, Roth B, Gustafsson R, Ohlsson B (2019). Irregular dietary habits with a high intake of cereals and sweets are associated with more severe gastrointestinal symptoms in IBS patients. Nutrients.

[46] Nordic Council of Ministers. Nordic Nutrition Recommendations 2012—Integrating nutrition and physical activity (2012).

[47] Jakobsson U, Larsson C (2014). Smoking and chronic pain among people aged 65 years and older. Pain Pract..

[48] Brennan PL, Schutte KK, Moos RH (2005). Pain and use of alcohol to manage pain: Prevalence and 3-year outcomes among older problem and non-problem drinkers. Addiction.

[49] Senba E, Kami K (2017). A new aspect of chronic pain as a lifestyle-related disease. Neurobiol. Pain.

[50] Briggs AM (2011). Individuals with chronic low back pain have greater difficulty in engaging in positive lifestyle behaviours than those without back pain: An assessment of health literacy. BMC Musculoskelet. Disord..

[51] Ambrose KR, Golightly YM (2015). Physical exercise as non-pharmacological treatment of chronic pain: Why and when. Best Pract. Res. Clin. Rheumatol..

[52] Husak AJ, Bair MJ (2020). Chronic pain and sleep disturbances: A pragmatic review of their relationships, comorbidities, and treatments. Pain Med..

[53] O'Brien KM (2018). Telephone-based weight loss support for patients with knee osteoarthritis: A pragmatic randomised controlled trial. Osteoarthr. Cartil..

[54] Williams A (2018). Effectiveness of a healthy lifestyle intervention for chronic low back pain: A randomised controlled trial. Pain.

[55] Larsson B, Gard G, Karlsson L, Persson AL (2016). Patient expectations for a multimodal pain rehabilitation programme: Active participation and coping skills. A qualitative study. Disabil. Rehabil..

[56] Michie S, van Stralen MM, West R (2011). The behaviour change wheel: A new method for characterising and designing behaviour change interventions. Implement. Sci..

[57] Hollis JL (2021). The impact of healthy conversation skills training on health professionals' barriers to having behaviour change conversations: A pre-post survey using the theoretical domains framework. BMC Health Serv. Res..

[58] Parchment A (2021). Making every contact count and healthy conversation skills as very brief or brief behaviour change interventions: A scoping review. J. Public Health.

[59] Amy Janke E, Kozak AT (2012). The more pain I have, the more I want to eat": Obesity in the context of chronic pain. Obesity (Silver Spring).

