# Supplementary material for: Eating habits and the desire to eat healthier among patients with chronic pain: a registry-based study
Source: Sci Rep. 2024 Feb 27;14:4705. doi: 10.1038/s41598-024-55449-z (PMC10897138; doi:10.1038/s41598-024-55449-z)
Supplement: Supplementary file 1 — Supplementary Information. [file 41598_2024_55449_MOESM1_ESM.docx]

# **Eating Habits and the Desire to Eat Healthier among Patients with Chronic Pain: A Registry-based Study**

**Huan-Ji Dong, Katherine Brain, Max Olsson, Elena Dragioti, Björn Gerdle, Bijar Ghafouri**

# Supplemental document: analysis of missing data

As shown in Table S1, we performed the analysis of missing data patterns. A total of 1360 values (4.9%) were missing and 697 (32.4%) of the 2152 cases contained at least one missing value. The missing cases were excluded from the original multivariable regression analysis.

**Table SI** missing data

|  | Missing | | Valid N |
| --- | --- | --- | --- |
|  | N | Percent |  |
| Pain duration | 307 | 14.3% | 1845 |
| LISAT-economy | 219 | 10.2% | 1933 |
| BMI-category | 173 | 8.0% | 1979 |
| Pain regional index | 129 | 6.0% | 2023 |
| Emotional distress (HADS-total) | 122 | 5.7% | 2030 |
| Frequency of alcohol consumption | 96 | 4.5% | 2056 |
| Frequency of confectionery consumption | 70 | 3.3% | 2082 |
| Tobacco use | 67 | 3.1% | 2085 |
| Frequency of Vegetables and fruits intake | 63 | 2.9% | 2089 |
| Regularity of mealtimes | 59 | 2.7% | 2093 |
| Frequency of fast-food consumption | 55 | 2.6% | 2097 |
| Gender | 0 | 0% | 2152 |
| Age | 0 | 0% | 2152 |

By excluding the two variables containing most missing cases (>10%) — pain duration and LISAT-economy, we performed the multivariable regression model (forward LR). The significant variables in the original model were still significant in the new model (Table S2). A total of 450 cases (20.1%) contained at least one missing value.

**Table SII** Multivariate regression model excluding Pain Duration and LiSAT-enonomy

|  | Variables associated with desired healthier eating,  Odds Ratio (95% Confidence Interval) |
| --- | --- |
|  |  |
| Regularity of mealtimes | 0.76 (0.67 - 0.86) |
| Frequency of Vegetables and fruits intake | 0.80 (0.72 - 0.89) |
| Frequency of confectionery consumption | 1.18 (1.01 - 1.38) |
| Frequency of fast-food consumption | 1.51 (1.21 - 1.90) |
| Age (55+ y, reference category) |  |
| 18-29 years | 1.94 (1.30 - 2.90) |
| 30-54 years | 1.49 (1.07 - 2.07) |
| Pain regional index | 1.02 (1.01 - 1.03) |
| Obesity | 1.34 (1.02 - 1.76) |

Another method to evaluate the effect on the results of these missing data is to apply multiple imputation. The literature describes three types of missing data: missing completely at random (MCAR), missing at random (MAR), and missing not at random (MNAR) ^1^. Traditional statistical analysis such as linear regression and binary logistic regression is based on the assumption of MCAR. However, most clinical epidemiological research is neither MCAR nor MNAR but MAR ^2^. Multiple imputation (MI) can be used to handle missing data under the MAR assumption, with a purpose of providing unbiased and valid estimates of associations based on information from the available data ^1,3^. MI consisted of 3 steps: data imputation (i.e. multiple imputation by chained equations (MICE)), analysis of each imputed data set and a final estimate of odds ratios in the combination of all the imputed data sets (pooled) ^3^.

Multiple imputation was applied using m =100 (iterations =50) imputed data set with predictive mean matching method and included all the variables of interest (predictors and outcomes). Several arguments and suggestions on the number of imputations have been discussed previously ^2,4,5^. Using the pooled sample, we found that the regression model did not in fact change much as a result of imputing missing data (Table S3). We noted a slightly higher odds ratio of obesity category than the original study sample.

In clinical practice as well in obesity research, we are aware of the consequence of weight stigma and bias ^6,7^. Patients who did not report body weight might not simply be missing completely at random (MCAR) or missing at random (MAR). However, multiple imputation method may handle both MCAR and MNAR ^2,8^. In line with some other studies, the missing BMI data does not appear likely to have impacted conclusions from a traditional complete case analysis of these data ^9,10^.

**Table S3** Multivariate logistic regression model using pooled data after multiple imputation (m=100, iterations=50)

|  | Variables associated with desired healthier eating,  Odds Ratio (95% Confidence Interval) |
| --- | --- |
|  |  |
| Regularity of mealtimes | 0.78 (0.69 - 0.87) |
| Frequency of Vegetables and fruits intake | 0.84 (0.76 - 0.92) |
| Frequency of candy consumption | 1.18 (1.03 - 1.35) |
| Frequency of fast-food consumption | 1.51 (1.24 - 1.85) |
| Age groups (55+ y, reference category) |  |
| 18-29 y | 1.97 (1.38 - 2.81) |
| 30-54 y | 1.37 (1.03 - 1.83) |
| Pain regional index | 1.02 (1.01 - 1.03) |
| Obesity | 1.51 (1.19 - 1.92) |

REFERENCES

1 Rubin, D. B. Inference and missing data. *Biometrika* **63**, 581-592 (1976). https://doi.org:10.1093/biomet/63.3.581

2 Pedersen, A. B. *et al.* Missing data and multiple imputation in clinical epidemiological research. *Clin Epidemiol* **9**, 157-166 (2017). https://doi.org:10.2147/CLEP.S129785

3 Harel, O. *et al.* Multiple Imputation for Incomplete Data in Epidemiologic Studies. *American Journal of Epidemiology* **187**, 576-584 (2017). https://doi.org:10.1093/aje/kwx349

4 Stuart, E. A., Azur, M., Frangakis, C. & Leaf, P. Multiple imputation with large data sets: a case study of the Children's Mental Health Initiative. *American journal of epidemiology* **169**, 1133-1139 (2009). https://doi.org:10.1093/aje/kwp026

5 Heymans, M. W. E., Iris. (Heymans and Eekhout, Amsterdam, 2019).

6 Prunty, A., Hahn, A., O’Shea, A., Edmonds, S. & Clark, M. K. Associations among enacted weight stigma, weight self-stigma, and multiple physical health outcomes, healthcare utilization, and selected health behaviors. *International Journal of Obesity* (2022). https://doi.org:10.1038/s41366-022-01233-w

7 Fruh, S. M. *et al.* Obesity Stigma and Bias. *J Nurse Pract* **12**, 425-432 (2016). https://doi.org:10.1016/j.nurpra.2016.05.013

8 Van Buuren, S. *Flexible imputation of missing data*. (CRC press, 2018).

9 Razzaghi, H. *et al.* Impact of Missing Data for Body Mass Index in an Epidemiologic Study. *Matern Child Health J* **20**, 1497-1505 (2016). https://doi.org:10.1007/s10995-016-1948-6

10 Novotny, P. J. *et al.* Do Missing Values Influence Outcomes in a Cross-sectional Mail Survey? *Mayo Clin Proc Innov Qual Outcomes* **5**, 84-93 (2021). https://doi.org:10.1016/j.mayocpiqo.2020.09.006
